# Ossiculoplasty: A Prospective Study of 80 Cases

**Published:** 2014-07

**Authors:** Shrinivas Shripatrao Chavan, Prateek V Jain, Jeevan N Vedi, Dharmendra kumar Rai, Himayat Kadri

**Affiliations:** 1 *Department of Otorhinolaryngology, Government Medical College, Aurangabad, India.*

**Keywords:** Chronic suppurative otitis media, Ossiculoplasty, Ossicular involvement.

## Abstract

**Introduction:**

The use of ossicular graft material in ossicular chain reconstruction has significantly improved hearing results hearing after tympanoplasty and tympanomastoid surgery for chronic otitis media. Today, otologists have a wide array of tools from which to choose, but may find it difficult to know which middle ear implant works best.

**Materials and Methods:**

A prospective study of 80 patients who underwent ossiculoplasty was performed in the ear, nose, and throat (ENT) department at a tertiary health care facility from 2011 to 2013. Patients with chronic suppurative otitis media with an air-bone gap (ABG) of >25 dB with ossicular involvement were included in the study. Total ossicular replacement prosthesis (TORP), partial ossicular replacement prosthesis (PORP), and refashioned incus were used. Success was defined as ABG <25 dB on postoperative Day 90.

**Results:**

The majority patients were of middle age with moderate conductive hearing loss. Incus was the most susceptible ossicle. Overall success rate in this study was 80.0% with an average change of 15.76 dB in ABG.

**Conclusion:**

With continuing advances in our understanding of middle ear mechanics, the results of ossiculoplasty are improving and results can be very rewarding in experienced hands. Severity of preoperative ear discharge, preoperative mastoid cellularity, presence of disease, and surgical procedure proved to be significant prognostic factors. Autograft incus and PORP fared better when the malleus handle was present while TORP gave better results when the malleus handle was eroded.

## Introduction

One of the most intriguing topics in middle ear surgery is the reconstruction of the conductive mechanism. Ever since Matte’s first myringostapediopexy in 1901, there has been a quest for the ideal middle ear implant with the understanding that the middle ear environment in chronic ear disease is probably the main factor in determining treatment success (1,2). 

The plethora of prostheses available is witness to the ongoing research for the ’ultimate prosthesis’ – that is, one able to reproduce and perhaps improve upon the natural impedance matching system at key hearing frequencies with predictable results. 

Initially, autografts and allografts were the ossicular replacement material used most widely by otologists. However, because of the fear of prion disease (including Creutzfeldt-Jakob disease), use of allografts came to a near halt. The advent of various bio-inert prosthetic materials has witnessed a shift in favor of prosthetic implants over last few decades (3, 4). Today, the otologist has a wide array of middle ear implants from which to choose, but may find it difficult to know which works best.

It is clear, however, that optimal results in ossicular reconstruction depend not only on the qualities of the prosthesis, but also on the environment in which it is placed and the surgical techniques used. Unfortunately, the literature is rife with controversy concerning the middle ear factors and types of pathologic process that are most important in predicting outcome.

In this study, we investigate the disease process affecting the ossicular chain and propose improved patient selection criteria for ossiculoplasty in light of postoperative hearing assessment of patients, considering various prognostic factors.

## Materials and Methods

This prospective study is based upon a series of 80 patients who underwent ossiculoplasty at a tertiary health care facility with adequate outpatient, inpatient, and diagnostic capabilities during the period from July 2011 to August 2013. Patients aged 15–70 years with chronic suppurative otitis media (CSOM) with a air bone gap (ABG) of >25 dB and ossicular involvement were included in the study.

Patients with sensorineural hearing loss, complicated CSOM, normal tympanic membrane, revision surgery, or a fixed stapes footplate were excluded from the study.

All patients were first seen in the outpatient department, where a detailed history and a thorough general and ear, nose, and throat (ENT) examination was performed. Emphasis was given to otological examin- ation including otomicroscopy and tuning-fork test. Each patient underwent a pure tone audiometry (PTA) assessment for subjective assessment of the hearing loss. The air conduction threshold, bone conduction threshold, and ABG was calculated conside ring the hearing threshold at 500 Hz, 1000 Hz, and 2000 Hz (5).

The patients were administered intravenous (IV) antibiotics for a minimum of 1 day prior to the operative procedure. The mastoid region was shaved for proper access to the operative area. All patients were operated on under general anesthesia using a postauricular approach to allow a consistent intraoperative environment across patients. The decision to perform mastoidectomy was taken after noting the intraoperative findings by the operating surgeon. Autograft incus and Teflon partial ossicular replacement prosthesis (PORP) were used for ossiculoplasty if a stapes suprastructure was present. Teflon total ossicular replacement prosthesis (TORP) was used when the stapes suprastructure was absent. The tympanic membrane was reconstructed by underlay technique using a temporalis fascia graft. A thin slice of cartilage was placed between the prosthesis and temporalis fascia graft if the handle of the malleus was involved. 

Patients were maintained on IV antibiotics, oral antihistamines, and analgesic therapy for 2 days in the ward. The mastoid dressing was changed after 48 hours. The patient was discharged on the third day with oral antibiotics, analgesic therapy, and antihistamines.

Postoperative dizziness was observed in 10 patients and was managed using labyrinthine sedatives. Nystagmus was observed in five patients, and loss of taste sensation was seen three patients. Facial palsy was not seen in the postoperative period in any patient. Ossicular graft extrusion was evident in six patients.

Patients were instructed to keep the ear dry, avoid sneezing, and travelling at high and low altitude.

All patients were asked to return for follow-up on Days 7, 15, and 45 after surgery, as well as after 3 and 6 months. On Day 7, the sutures were removed and topical administration of a medicated ear drop was advised.

At follow-up, the patients were asked about ear discharge, hearing improvement, giddiness, and upper respiratory infections. 

A thorough ENT examination was performed in order to detect residual perforation of the tympanic membrane, ear discharge, or other nose or throat infections on Day 15 after removing the remaining Gelfoam. Tuning-fork tests were performed and PTA was performed on Days 45 and 90 to assess hearing improvement. 

Successful surgery was defined as a postoperative ABG of <25 dB (6) on postoperative Day 90 (1,7). Results were analyzed using the Chi-square test and Fisher’s exact test.

## Results

In this descriptive, analytical prospective study, a total of 80 cases of ossiculoplasty were studied in detail. The mean age at which patients were operated on was 34 years and 3 months, with males constituting 52.5% of patients. Decreased hearing was the most common presenting symptom followed by ear discharge. According to the severity of ear discharge, the cases were divided into four groups: no discharge; minimal discharge (discharge accumulating in the external auditory canal [EAC] but not soiling linen at night); intermediate discharge (discharge soiling linen at night); and profuse discharge (discharge reappea- ring immediately after cleaning the ear).

**Table 1:** Factors affecting ossiculoplasty

**No.**		**Factor**	**No. cases**	**Success rate**	**P-value**
1	Severity of ear discharge	No discharge	02	100%	0.0350
		Minimal discharge*	26	92.30%	
		Intermediate discharge**	36	80.55%	
		Profuse discharge***	16	56.25%	
2	Material used	Autograft Incus	27	85.18%	0.6789
		Teflon PORP	24	79.16%	
		Teflon TORP	29	75.86%	
3	Middle ear mucosa	Normal	61	83.60%	0.1904
		Diseased	19	68.42%	
4	Mastoid cellularity	Pneumatic	45	88.88%	0.0496
		Sclerotic	35	68.57%	
5	Cholesteatoma	Present	36	69.44%	0.0986
		Absent	44	88.63%	
6	Mastoidectomy	No	41	92.68%	0.0135
		Canal wall down	34	67.64%	
		Canal wall up	05	60.00%	
7	Handle of malleus	Present	71	84.50%	0.0135
		Absent	09	44.44%	
8	Stapes suprastructure	Present	51	82.35%	0.5650
		Absent	29	75.86%	

* Discharge accumulating in EAC but not soiling linen at night;

** Discharge soiling linen at night;

*** Discharge reappearing immediately after cleaning the ear

**Fig 1:**
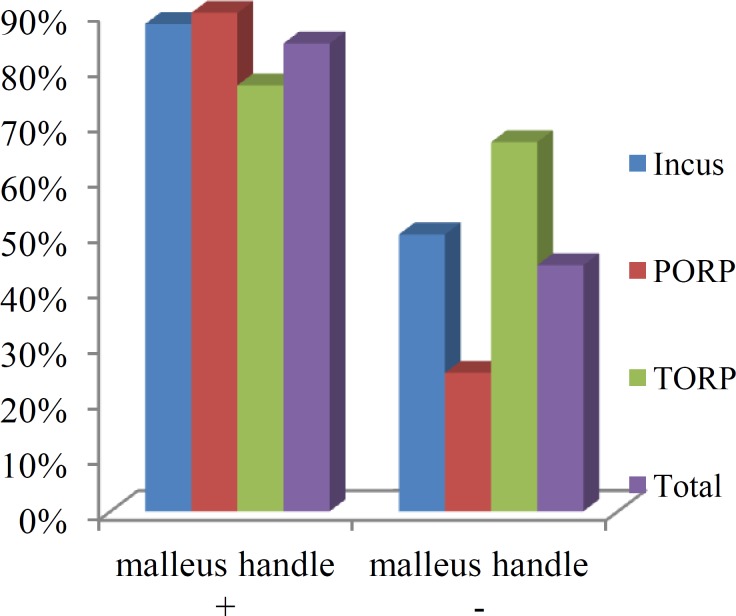
Efficacy of different materials when the malleus handle was affected

Mean preoperative air conduction (AC) was 47.89 dB, mean bone conduction (BC) was 13.35 dB, and mean ABG was 34.54 dB. Middle ear mucosa was diseased (polypoidal or granulations) in 19 patients. Pneumatic mastoid cavity was present in 43.75% patients. The long process of incus, associated with 74 patients, was the part of the ossicular chain most commonly involved, followed by the stapes supras- tructure in 29 patients. The malleus was the most resistant ossicle to the disease process, involved in 10 patients. Cholesteatoma was evident in 45.0% of the patients. Teflon TORP was the most commonly used prosthetic material (29 patients, 36.25%), followed by refashioned incus (27 patients, 33.75%) and Teflon PORP (24, 30.0%). No mastoidectomy was performed in 41 patients (51.25%), while canal-wall-down mastoidectomy was performed in 34 patients (42.5%). Canal-wall-up mastoidec- tomy was performed in five patients (6.25%). 

The mean change in ABG was 15.76 dB in 80 patients. Severity of preoperative discharge, preoperative mastoid cellularity, mastoidectomy, and presence of handle of malleus were the factors found to significantly affect the result of ossiculo- plasty (Table. 1).

When the malleus handle was present, PORP and incus gave better results than TORP. When the malleus handle was eroded, the results were superior when the stapes suprastructure was also involved and TORP was used as an ossicular replace- ment material compared with when the stapes suprastructure was present (Fig.1).

## Discussion

Current techniques of ossiculoplasty have generally evolved empirically as a result of trial and error. With the evolution of newer surgical techniques and advances in the instrument armamentarium available to the otologist, the hearing outcome of ossiculoplasty has shown a noticeable improvement over recent years. Success in ossiculoplasty is determined by technical ability and, to a large extent, case selection. Likewise, much of the variability in the literature concerning hearing results after ossiculoplasty is due to a lack of understanding and uniform reporting of those middle ear factors that influence the results. Importance of a middle ear grading system that is reliable and simple to use cannot be overemphasized. Valid attempts have been made to elucidate prognostic factors in ossiculoplasty, each contributing significantly to our understanding of middle ear disease and its effect on hearing results with ossiculoplasty.

Ossicular grafts and prostheses must couple well at their ends to bone or soft tissue, but must remain suspended in air elsewhere in order to transmit sound effectively. Additionally, ossicular implants are subject to resorption from persistent or recurrent infection and extrusion from negative pressure and tubal insufficiency. In the case of homograft and synthetic prostheses, they are also potentially subject to immune-mediated rejection.

In the present study, the mean age at presentation was approximately 34 years and 3 months, with the patients ranging from 15 to 62 years of age. There was a slight male predominance, with a male:female ratio of 1.10:1.

Decreased hearing was the most common complaint among patients (100%), followed by ear discharge (97.5%) in this study. A minority of patients also complained of tinnitus (15.0%), earache (3.75%), and vertigo (5.0%). The right ear was involved in 42 patients, while the left ear was involved in 48.75% cases. 

In the present study, success was defined as an ABG <25 dB on postoperative Day 90 (1,6,7). Out of 80 cases, 64 patients had an ABG <25 dB, accounting for an overall success rate of 80.0%. Five different surgeons operated on the patients and no statistically significant difference was found on success rates achieved according to operating surgeon (73.7–87.0%). The average change in ABG was 15.76 dB across 80 patients. 

A clear trend for a worsening of outcome was observed as the severity of discharge increased, while the condition of middle ear mucosa did not significantly affect the success rate of ossiculoplasty. These findings correlate well with those of Bellucci (8), who classified all cases into those with a good prognosis (Group 1; i.e. never infected), a fair prognosis (Group 2; i.e. intermittent discharge), a poor prognosis (Group 3; i.e. unremitting discharge), and a very poor prognosis (Group 4; i.e. cleft palate and nasopharyngeal deformities) according to the degree of otorrhea and Eustachian tube function. Dornhoffer did not find the above Bellucci classification to be statistically significant (2); however he found a clear trend for worsening outcome as he moved from Group 1 to Group 3. He also found the middle ear mucosa to affect the outcome of the surgery. Finally, he reported a trend towards worsening hearing results with mucosal thickening, but statistically significant results were obtained only when fibrosis was present.

Mastoid cellularity had a significant effect on the outcome of the surgery. In the presence of cholesteatoma, poor results were seen when the stapes suprastructure or malleus handle were affected (55.6% and 40.0% respectively). In contrast, in patients with cholesteatoma, when the stapes suprastructure was present, the success rate was 74.1%, and 74.2% when the malleus handle was present. It was the effect of cholesteatoma on ossicles rather than the cholesteatoma itself that affected the result of ossiculoplasty.

The pathologic condition of the middle ear as a predictor of outcome is a very confusing issue in the literature. Dornhoffer reported that the pathologic condition associated with the surgical indication was not significant (2). He found that the mucosal status and presence of drainage were more significant than the pathologic conditions initiating the surgical procedure. According to this report, it was not the presence of cholesteatoma, but the associated middle ear disease that was found to be significant. 

 Black found the middle ear mucosa to be a predictor of postoperative hearing outcome (9). He reported his SPITE method of assessment, which divided prognostic factors into surgical, prosthetic, infection, tissue, and Eustachian factors.

Albu et al also found significance in separating samples into simple otitis media (10), granulating otitis media,and cholesteatoma in relation to worsening results, although Brackmann et al found no significance in this regard (11). 

Proliferation of fibrous tissue and the formation of adhesions are substantial problems that are more prone to occur when the middle ear mucosa is diseased, removed, or traumatized. Many different materials have been placed in the middle ear in an attempt to prevent formation of adhesions and fibrous tissue. These materials include absorbable gelatin sponge (Gelfoam), hyaluronic acid, Silastic, and Teflon. The ideal material would remain in place for several weeks to allow sufficient time for mucosal regeneration and would then undergo degradation and resorption so that the ear may become aerated without fibrosis. None of the currently available spacer materials is ideal, although Gelfoam seems to be the better spacer material amongst the choices available at present (12).

In the present study, the long process of incus was the most susceptible part of the ossicular chain, affected due to the disease process in 74 patients, followed by the stapes in 29 patients with the malleus being the most resistant amongst the three, being affected in only 10 cases. This correlates with the precarious blood supply to the long process of incus that results in the incus being the most susceptible ossicle for erosion (13).

The presence of a malleus handle contributed to a successful surgical outcome, but the presence of a stapes suprastructure did not significantly affect the outcome of the surgery. When the malleus handle was present, PORP and incus gave better results than TORP. When the malleus handle was eroded, the results were superior when the stapes suprastructure was also involved and TORP was used as an ossicular replacement material than when the stapes suprastructure was present.

The status of the ossicular chain as a determinant of hearing results has been somewhat controversial in the literature. Theoretically, the stapes superstructure should contribute little or nothing to the acoustic gain of the middle ear mechanism, whereas in fact the malleus actually may be significant acoustically through its action as a catenary lever and impedance matcher (14). Mishiro et al in his review of 720 patients (15), reported that both the stapes suprastructure and the malleus handle were significant in predicting the outcome of ossiculoplasty. However, he found the mobility of the stapes footplate to be an insignificant factor.

This study is consistent with that of Yung et al who found that the malleus handle was the only significant factor to determine the outcome of ossiculoplasty in the long run(1).

Dornhoffer et al (2), similarly found only the malleus manubrium to be significant, whereas the stapes superstructure contributed little. Interestingly, he found that the presence of the stapes superstructure was detrimental in cases involving more severe mucosal fibrosis. Poorer hearing results occurred in those cases where the stapes was present and the malleus was absent. This is consistent with the present study.

In contrast, Brackmann et al and Goldenberg found the contribution of the malleus handle to be insignificant (12,16).

A wide variety of autografts, homografts, synthetic ossicular grafts, and prostheses have been employed for reconstructing the ossicular chain. With a number of prosthesis being available, comparisons become inevitable. The ideal prosthesis for ossiculo- plasty should be compatible, stable, safe, readily available, easily insertable, and capable of yielding optimal sound transmission. There was no statistical difference found in the use of different types of ossicular implants for ossiculoplasty, i.e refashioned incus, Teflon PORP, or TORP.

Jha et al concluded that cartilage (17), bone, and gold are better and more cost effective alternatives to plastipore and titanium. As in this study, Yung et al found no difference between the different types of prostheses used (1). However, in contrast with present study, Jackson et al achieved better results with Teflon TORP than PORP in his study of 141 cases of ossiculoplasty (18).

In the present study, the type of mastoidectomy affected the outcome of ossiculoplasty, with canal-wall-up and down mastoidectomy providing poorer results as compared with no mastoidectomy. The outcome when canal-wall-up mastoidectomy was performed was poorer as compared with those undergoing canal-wall-down mastoidectomy; probably due to the smaller number of cases undergoing canal-wall-up mastoidectomy as compared with canal-wall-up procedures (5 vs. 34, respectively). 

Conceptually, one might expect poorer hearing results in a canal-wall-down situation because a shallow middle ear cleft is less acoustically efficient and preservation or reconstruction of the canal wall favors a more physiologic ossiculoplasty, with less chance for contact and fibrosis of the prosthesis to the promontory or facial nerve. This is consistent with the findings of Albu et al (11), who showed a detrimental association with the performance of a mastoidectomy.

Dornhoffer et al stated that the type or complexity of the surgical procedure had a significant impact on the hearing results, both in the performance of a mastoidectomy with the surgical procedure and more importantly, in the removal of the canal wall. He advocated partial mastoid obliteration and reconstruction of the tympanic ring with cartilage when performing canal-wall-down surgery in an attempt to deepen the middle ear cleft, and reported encouraging hearing results (2,19).

The detrimental impact of removing the canal wall has not, however, been universally shown, as in the study conducted by Brackmann et al (12).

## Conclusion 

The incus was the most susceptible ossicle to the disease process, whereas the malleus was the most resistant.

Gelfoam appears to be an optimal spacer material amongst those currently available.

Less severe ear discharge preoperatively, pneumatic mastoid capacity, no requirement for mastoidectomy, and presence of the handle of malleus indicated a favorable outcome of ossiculoplasty.

Autograft incus and PORP fared better when the malleus handle was present, while TORP gave better results when the malleus handle was eroded.

With the continuing advances in our understanding of middle ear mechanics, the outcomes of ossiculoplasty are improving. By paying careful attention to the principles of ossicular reconstruction and the lessons learned from basic science as translated to clinical practice, surgeons are increasingly able to optimize hearing results for their patients.
